# NIPA (Nuclear Interaction Partner of ALK) Is Crucial for Effective NPM-ALK Mediated Lymphomagenesis

**DOI:** 10.3389/fonc.2022.875117

**Published:** 2022-05-13

**Authors:** Stefanie Kreutmair, Lena Johanna Lippert, Cathrin Klingeberg, Corinna Albers-Leischner, Salome Yacob, Valeria Shlyakhto, Tony Mueller, Alina Mueller-Rudorf, Chuanjiang Yu, Sivahari Prasad Gorantla, Cornelius Miething, Justus Duyster, Anna Lena Illert

**Affiliations:** ^1^ Department of Internal Medicine I, Medical Center-University of Freiburg, Faculty of Medicine, University of Freiburg, Freiburg, Germany; ^2^ German Cancer Consortium and German Cancer Research Center, Heidelberg, Germany; ^3^ Department of Infectious Diseases and Respiratory Medicine, Charité - Universitätsmedizin Berlin, Berlin, Germany; ^4^ Department of Hematology, Oncology and Bone Marrow Transplantation with Section Pneumology, Hubertus Wald Comprehensive Cancer Center Hamburg, University Medical Center Hamburg-Eppendorf, Hamburg, Germany; ^5^ Department of Tumor Biology, University Medical Center Hamburg-Eppendorf, Hamburg, Germany; ^6^ Department I of Internal Medicine, Center for Molecular Medicine Cologne (CMMC), University of Cologne, Cologne, Germany; ^7^ Department of Hematology and Oncology, Medical Center, University of Schleswig-Holstein, Lübeck, Germany

**Keywords:** NIPA, NPM-ALK, anaplastic large cell lymphoma, lymphomagenesis, transplantation mouse model

## Abstract

The NPM-ALK fusion kinase is expressed in 60% of systemic anaplastic large-cell lymphomas (ALCL). A Nuclear Interaction Partner of ALK (NIPA) was identified as a binding partner of NPM-ALK. To identify the precise role of NIPA for NPM-ALK-driven lymphomagenesis, we investigated various NPM-ALK^+^ cell lines and mouse models. *Nipa* deletion in primary mouse embryonic fibroblasts resulted in reduced transformation ability and colony formation upon NPM-ALK expression. Downregulating NIPA in murine NPM-ALK^+^ Ba/F3 and human ALCL cells decreased their proliferation ability and demonstrated synergistic effects of ALK inhibition and NIPA knockdown. Comprehensive *in vivo* analyses using short- and long-latency transplantation mouse models with NPM-ALK^+^ bone marrow (BM) revealed that *Nipa* deletion inhibited NPM-ALK-induced tumorigenesis with prolonged survival and reduced spleen colonies. To avoid off-target effects, we combined *Nipa* deletion and NPM-ALK expression exclusively in T cells using a lineage-restricted murine ALCL-like model resembling human disease: control mice died from neoplastic T-cell infiltration, whereas mice transplanted with *Lck-Cre^TG/wt^Nipa^flox/flox^* NPM-ALK^+^ BM showed significantly prolonged survival. Immunophenotypic analyses indicated a characteristic ALCL-like phenotype in all recipients but revealed fewer “stem-cell-like” features of *Nipa-*deficient lymphomas compared to controls. Our results identify NIPA as a crucial player in effective NPM-ALK-driven ALCL-like disease in clinically relevant murine and cell-based models.

## Introduction

Anaplastic large cell lymphoma (ALCL) is an aggressive peripheral T-cell non-Hodgkin’s lymphoma, usually presenting at advanced stages as a systemic disease with multi-nodal involvement. It is characterized by anaplastic morphology, expression of CD30 (Ki-1), and a cohesive growth pattern with infiltration of lymph node sinuses (1, 2). Typical T-cell markers are rarely detectable in ALCL; about 40% have a so-called null-cell phenotype as they lack both T-cell marker and receptor rearrangements (2–4). A t (2,5)(p23;q35) translocation leads to the expression of the chimeric Nucleophosmin-anaplastic lymphoma kinase (NPM-ALK) in approximately 60% of systemic ALCL cases (5). Through NPM-mediated homodimerization, ALK is constitutively activated in ALCL, leading to increased proliferation and tumorigenesis through promitogenic, antiapoptotic, and transforming pathways, particularly STAT3, JUNB, AP-1, MAPKs, and PI3K/mTOR/AKT (6–12). Various animal models have demonstrated the essential role of NPM-ALK in ALCL pathogenesis (13–18), yet the key pathway for lymphomagenesis remains to be identified.

ALK^+^ ALCL is diagnosed mainly in children and young adults, whereas the ALK-negative form is more common in older adults (19, 20). Although initial responses to standard chemotherapy regimens are frequently observed in ALK^+^ ALCL, many patients relapse within five years or later, which is particularly relevant given the young age of onset. Studies with ALK-inhibitors and CD30-specific antibodies have given promising results; however, the need for targeted therapies is high (21–27).

Dysregulation of F-box protein mediated ubiquitylation is involved in the pathogenesis of many diseases. To date, 69 F-box proteins have been identified as fulfilling crucial functions in carcinogenesis, such as FBXW7 in T-ALL or Burkitt lymphoma, FBXO11 in DLBLC, or FBXO25 in MCL (28–30). They can function as tumor suppressors and oncoproteins, thus being promising therapeutic approaches, although difficult to target. The F-box protein Nuclear Interaction Partner of ALK (NIPA, ZC3HC1, HSPC216) was first identified in 2003 by Ouyang et al. in a yeast-two hybrid screen as being constitutively phosphorylated in NPM-ALK-positive cells (31). NIPA defines an oscillating ubiquitin E3 ligase as part of an SKP1-ROC1-CUL1^F-BOX^ (SCF)-complex that targets nuclear cyclin B1 in interphase. At the G_2_/M transition, NIPA dissolves from the SCF^NIPA^-complex after ERK2 phosphorylation at serin-354, allowing cyclin B1 to accumulate in the nucleus and mitosis to occur (32, 33). *Nipa*-deficient mice are viable but sterile due to impaired homologous chromosomal pairing in meiosis (34). Recent studies have shown that NIPA plays a pivotal role in the murine hematopoietic stem cell (HSC) pool. As a Fanconi anemia-associated protein, NIPA regulates the nuclear abundance of FANCD2, thereby making it essential for a functional DNA repair/Fanconi anemia/BRCA pathway. Aging or other replication stress triggers the decrease and functional decline of *Nipa*-deficient HSCs, resulting in complete bone marrow failure (35).

The role of NIPA in NPM ALK-positive ALCL and the functional consequences of the NIPA–NPM-ALK interaction remain unclear. Although it has been shown that the tyrosine kinase NPM-ALK does not directly phosphorylate NIPA (31), co-expression of NIPA and NPM-ALK results in constitutive NIPA phosphorylation at five S/T key residues located in the ALK-binding domain (Ser-338, Ser-344, Ser-370, Ser-381, and Thr-387), which were recently shown to be crucial for NIPA–NPM-ALK binding capacity and may be involved in ALK-localization (36). NPM-ALK-mediated NIPA phosphorylation has no effect on the cell cycle rate of Ba/F3 cells and mouse embryonic fibroblasts (MEFs), nor does it change the structure of the SCF^NIPA^-complex structure, implying that this phosphorylation activates a cell-cycle-independent function of NIPA (36).

In this study, we further investigated the effect of *Nipa* deficiency on NPM-ALK mediated cell proliferation and transformation by using NPM-ALK positive cell lines and *Nipa* deficient ALCL mouse models. Since NIPA seems to be relevant for physiologic mitotic timing and DNA damage repair, its absence bears potential for both malignant transformation and apoptosis.

## Materials and Methods

### Constructs, Cell Culture, and Virus Generation

For virus production, Phoenix E ecotropic packaging cells (a kind gift from G. Nolan, Stanford, CA) were transiently transfected with the retroviral construct MSCV-STOP-NPM-ALK-IRES-EGFP (MSNAIE), Mig^NPM-ALK^, pBABE-puroR^Nipa^, and viral supernatants were collected as described previously (14, 17, 18, 37). Retroviral titers were determined by the transduction of NIH/3T3 cells (DSMZ) as described previously (17).

### MTS Assay and Soft Agar Assay

The assays were performed as previously described (36). In brief, retrovirally infected Ba/F3 or Karpas299 cells express NPM-ALK and miR^ctrl^ [as previously described (38)] or miR^NIPA^ (5’-TGC TGT TGA CAG TGA GCG CTC CAT TGG AAT CCA CAA GCA ATA GTG AAG CCA CAG ATG TAT TGC TTG TGG ATT CCA ATG GAA TGC CTA CTG CCT CGG A-3’) were plated on 96-well plates in triplicates (5,000 cells in 100 µl of RPMI). To assess cell proliferation, MTS reagent (Promega, Madison, WI, USA) was added to the cells at the indicated time points and incubated for 2 h at 37°C. Extinction at 492 nm was measured using a microplate reader (Tecan, Männedorf, Switzerland).

For soft agar proliferation assays, we prepared primary *Nipa^+/+^* or *Nipa^−/−^* MEFs from embryos (E13.5) and cultured them in DMEM (PAA Laboratories) supplemented with 15% FCS under low oxygen conditions. Early passages only were used for the indicated experiments. *Nipa^ko/ko^* MEFs were retrovirally infected with vectors containing NPM-ALK and Flag-NIPA wt. The assay was performed as previously described (39). A total of 25,000 and 100,000 cells were plated in soft agar in 6-well plates. Colonies were counted between days 15 and 20 after plating.

### EdU Cell Cycle Assay

For cell cycle measurements, EdU along with FxCycleViolet, was used according to the instructions of the manufacturer.

### Immunoblot

Immunoblotting was performed as described previously (33, 34). Antibodies against βACTIN (A5316) and NIPA (ZC3HC1, HPA024023) were purchased from Sigma. ALK (cs-3333) was purchased from Cell Signaling, GAPDH (OSG-00033G) from Osenses. Quantification of immunoblots was performed using LabImage 1D L340 software (Intas Science Imaging, Goettingen, Germany).

### Reagents

Recombinant murine Interleukin-3 (IL-3), IL-6, and SCF were purchased from R&D Systems (Minneapolis, MN, USA). Fetal calf serum, 5-Flouorouracil, and Polybrene were purchased from Sigma-Aldrich (St. Louis, MO, USA). DMEM (Dulbecco’s Modified Eagle Medium) and ES cell FBS (fetal bovine serum) were purchased from Thermo Scientific (Waltham, USA). Lipofectamine 2000 was purchased from Invitrogen (Carlsbad, CA, USA). TAE-684 was purchased from Axon Medchem (Groningen, NL).

### Mice

The Lck-Cre mouse line (B6.Cg-Tg(Lck-cre)548Jxm/J) was obtained from the Jackson Laboratory (Bar Harbor, Maine, USA). *Nipa* ^flox/flox^ mice were generated using a conditional knockout strategy (34). To achieve tissue-specific *Nipa* deletion, we crossed *Nipa* ^flox/flox^ mice with *Lck-Cre* transgenic mice. Littermates or age- and sex-matched mice were used as controls. All mice were backcrossed to a C57BL/6 background for more than ten generations. The animals were housed in a special cage system with autoclaved food and acidified water at the University of Freiburg. All procedures were performed in accordance with national and institutional guidelines for animal care and experiments.

### Transplantation Assays

Murine bone marrow was collected from *Lck-Cre* wildtype *Nipa* ^ko/ko^ and *Nipa* ^wt/wt^ or Lck transgenic *Nipa* ^flox/flox^ and *Nipa* ^wt/wt^ mice and infected as described previously (14, 38, 40). Briefly, 12-week-old male donor mice were treated once with 5-Fluorouracil (150 mg/kg) on day - 4 and BM cells were harvested from the tibia and femur. After preincubation overnight in BM media (DMEM, 30% FBS, 10 ng/ml mIL-3, 10 ng/ml mIL-6, and 50 ng/ml mSCF), BM cells were infected with retroviral supernatant as described previously (41). The infection efficiency was determined by flow cytometric analysis of EGFP expression. Female C57BL/6 wild-type recipient mice were irradiated with 850 rad and transplanted with the indicated cells *via* the tail vein injection. Peripheral blood was taken at the indicated time points and WBCs were measured using an automated counter (ABC scil vet). Transplanted mice were monitored for signs of disease and sacrificed and analyzed based on clinical signs.

### Flow Cytometry Analysis

Flow cytometry analysis was performed as described (35). The BD LSR Fortessa (BD Biosciences, Heidelberg, Germany) was used for analysis. Antibodies used to stain cell surface proteins were anti-mouse CD4 (GK1.5), CD8a (53-6.7), CD11b (Mac-1, M1/70), CD25 (PC61.5), CD44 (IM7), CD45R/B220 (RA3-6B2), CD45 (30-F11), CD90.2 (THY1.2, 53-2.1), CD117 (c-KIT, 2B8), CD127 (IL-7Ra, A7R34), GR1 (Ly-6G, RB6-8C5), SCA1 (D7), and TER119 (TER119) and the corresponding isotypes, which were obtained from BD Biosciences or eBiosciences (Frankfurt am Main, Germany).

### Statistical Analysis

A two-sided Student’s t-test was used for statistical analyses. The mean ± standard deviation was analyzed as indicated. The survival curves were produced using a log-rank (Mantel–Cox) test. P-values were defined as *p <0.05, **p <0.01, ***p <0.001, and ****p <0.0001.

## Results

### NIPA Deficiency Impairs NPM-ALK Mediated Colony Formation and Viability *In Vitro*

**Figure 1:**
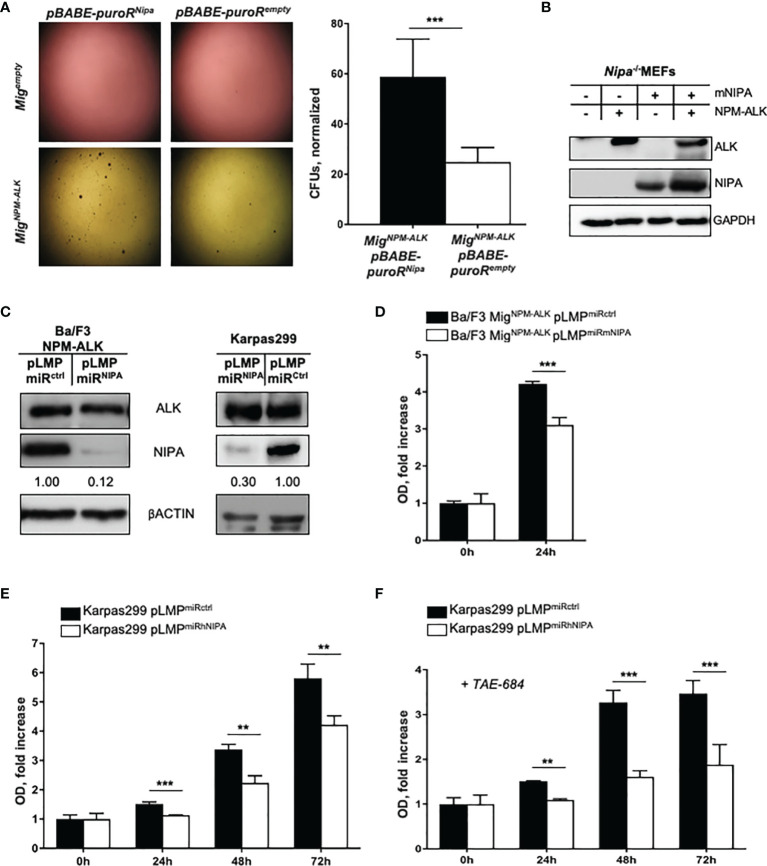
NIPA deficiency impairs NPM-ALK-mediated colony formation and viability *in vitro.* **(A)** Colony formation assay in soft agar of *Nipa^ko/ko^* MEFs retrovirally infected with pBABE-puroR^Nipa^ or pBABE-puroR^empty^ vector and Mig^NPM-ALK^ or Mig^empty^ vector. Seeding of 100,000 cells per well, representative wells shown 18 days after plating. Colonies (*Nipa^ko/ko^* pBABE-puroR^Nipa^ Mig^NPM-ALK^) = 58.81 ± 6.13. Colonies (Nipa^ko/ko^ pBABE-puroR^empty^ Mig^NPM-ALK^) = 28.87 ± 1.67. Results from three independent experiments performed in duplicates. Mig = MSCV-IRES-EGFP. **(B)** Immunoblot of *Nipa^ko/ko^* MEFs retrovirally infected with pBABE-puroR^Nipa^ or pBABE-puroR^empty^ vector, and Mig^NPM-ALK^ or Mig^empty^ vector ensured correct protein expression. **(C)** Immunoblot of Ba/F3-cells retrovirally infected with Mig^NPM-ALK^ and pLMP^miRmNIPA^ or pLMP^miRctrl^, and of Karpas299-cells retrovirally infected with pLMP^miRhNIPA^ or pLMP^miRctrl^. **(D)** MTS assay of Ba/F3-cells retrovirally infected with Mig^NPM-ALK^ and pLMP^miRmNIPA^ or pLMP^miRctrl^ after 24 hours of incubation. Representative experiment shown, results reproduced in four independent experiments performed in triplicates. **(E)** MTS assay of Karpas299-cells retrovirally infected with pLMP^miRhNIPA^ or pLMP^miRctrl^ after 24, 48, and 72 h of incubation at standard conditions. Representative experiment shown, results reproduced in eight independent experiments performed in triplicates. **(F)** MTS assay of Karpas299-cells retrovirally infected with pLMP^miRhNIPA^ or pLMP^miRctrl^, treated with 0.5 nM of ALK-inhibitor TAE-684, after 24, 48, and 72 h of incubation at standard conditions. Representative experiment shown, results reproduced in three independent experiments performed in triplicates. OD, optical density at 490 nm. *p < 0.05, **p < 0.01, ***p < 0.001. Data shown as mean +SD.

To analyze the impact of NIPA on NPM-ALK mediated transformation and colony formation, we performed *in vitro* soft agar assays. *Nipa* ^ko/ko^ mouse embryonic fibroblasts (MEFs) were retrovirally infected with NPM-ALK and NIPA or empty vector control. As seen in **Figure 1A**, *Nipa*-deficient MEFs infected with NPM-ALK showed significantly lower numbers and smaller sizes of colonies in soft agar assays than NIPA-re-expressing controls (28.9 colony forming units (CFUs) vs. 58.8 CFUs; p = 0.008). Western blotting revealed the correct expression of NPM-ALK and NIPA (**Figure 1B**). In the absence of NPM-ALK, no colony growth was observed in either group, suggesting that *Nipa* deficiency alone has no transformative potential in MEFs in soft agar assays.

Using targeted genetic approaches, an efficient and durable NIPA knockdown of more than 70% was achieved in the murine IL-3 dependent Ba/F3 cell line and the human ALCL cell line Karpas299 (**Figure 1C**). Upon efficient NIPA downregulation, Ba/F3 cells were retrovirally infected with NPM-ALK. Proliferation was assessed by MTS assays under IL-3 withdrawal, where the optical density (OD) reflects the metabolization of MTS reagent and thus the number of viable cells present. NIPA knockdown significantly impaired the proliferation of NPM-ALK-positive Ba/F3 and Karpas299 cells. Within 24 h, the number of viable NPM-ALK-positive Ba/F3 grew 4.2 fold, whereas only 3.1 fold in NIPA knockdown cells (**Figure 1D**). Two-dimensional cell cycle analyses revealed no differences in the cell cycle profile of NPM-ALK-positive cells upon NIPA knockdown, pointing to a cell-cycle-independent function of NIPA in dependence of NPM-ALK (**Supplementary Figures 1A, B**).

To expand our data to human cells, we designed NIPA siRNAs targeting the human NIPA mRNA and downregulated NIPA in the human ALCL cell line Karpas299. Effective NIPA downregulation resulted in a significantly reduced proliferation (75% compared to controls) of ALCL cells measured at numerous time points after seeding (**Figure 1E**). Interestingly, NIPA downregulation in Karpas299 cells showed a significantly higher susceptibility to the ALK inhibitor TAE-684 (**Figure 1F**), suggesting a possible synergistic effect of ALK inhibition and NIPA knockdown.

### Loss of NIPA Prolongs Survival in Short and Long Latency NPM-ALK Driven Murine Tumorigenesis

**Figure 2:**
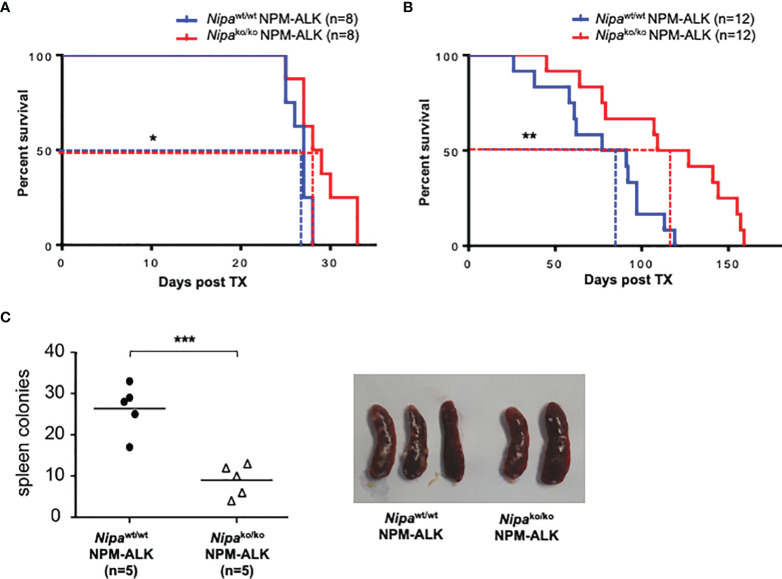
Loss of NIPA prolongs survival in short and long latency NPM-ALK driven murine tumorigenesis. **(A)** Kaplan–Meier survival curve of mice transplanted with 300,000 *Nipa^ko/ko^* and *Nipa^wt/wt^* bone marrow cells infected with Mig^NPM-ALK^ (3%). Median survival was 28.5 days (*Nipa^ko/ko^*, n = 8) vs. 27 days (*Nipa^wt/wt^*, n = 8). **(B)** Kaplan–Meier survival curve of mice transplanted with 200,000 *Nipa^ko/ko^* and *Nipa^wt/wt^* bone marrow cells infected with Mig^NPM-ALK^ (0.4%). Median survival was 118 days (*Nipa^ko/ko^*, n = 12) vs. 84 days (*Nipa^wt/wt^*, n = 12). **(C)** Number of spleen colonies in mice transplanted with *Nipa^ko/ko^* and *Nipa^wt/wt^* BMCs infected with Mig^NPM-ALK^ at final stage of disease. Mean amount of colonies per spleen were 10 (*Nipa^ko/ko^*, n = 5) vs. 28 (*Nipa^wt/wt^*, n = 5). Representative spleens shown. *p < 0.05, **p < 0.01, ***p < 0.001.

To analyze the impact of NIPA on NPM-ALK-induced tumorigenesis, we used a retroviral murine BM transplantation model for NPM-ALK-driven malignancies. As previously demonstrated by Miething et al. (17), transplantation of NPM-ALK-positive bone marrow cells (BMCs) in lethally irradiated recipient mice leads to two distinct phenotypes (polyclonal histiocytic malignancy vs. monoclonal B-lymphoid tumors), depending on disease latency. We performed analogous transplantation experiments using *Nipa* ^ko/ko^ and *Nipa* ^wt/wt^ donor BMCs. For the short latency model, mice were given 300,000 BMCs with 3.0% NPM-ALK (EGFP) positive cells. For the long latency model, 200,000 cells with 0.4% NPM-ALK (EGFP) positive cells were injected. Independent of the model used, mice transplanted with Mig^NPM-ALK^ *Nipa* ^ko^ *^/^* ^ko^ BMCs showed significantly prolonged survival with 28.5 vs. 27 days (p = 0.03) in short latency and 118 vs. 84 days (p = 0.008) in the long latency model, respectively (**Figures 2A, B**).

In the short latency model, the progression of the disease was furthermore assessed by the number of spleen colonies. Animals transplanted with Mig^NPM-ALK^ *Nipa* ^ko/ko^ bone marrow were found to have a significantly lower number of spleen colonies than controls, with 10 colonies per spleen vs. 28 in controls (p <0.001) (**Figure 2C**). However, the disease immunophenotype was not altered by the absence of NIPA in both the long and short latency models (data not shown). Taken together, our results highlight the crucial role of NIPA in NPM-ALK-driven tumorigenesis.

### Deletion of Nipa Delays Lymphoma Progression in an ALCL-Like Mouse Model

Based on these results, we hypothesized that NIPA influences NPM-ALK-driven transformation in an ALCL mouse model resembling the human clinical phenotype. This ALCL-like model is based on a lineage-specific Cre/LoxP-dependent expression of NPM-ALK by the retroviral construct MSCV-STOP-NPM-ALK-IRES-EGFP (MSNAIE) (**Supplementary Figure 2A**). Infection of LckCre^TG/wt^ BMCs with MSNAIE retrovirus and transplantation into lethally irradiated wild-type recipient mice leads to a systemic CD30-positive ALCL-like T-cell lymphoma (14, 18).

**Figure 3:**
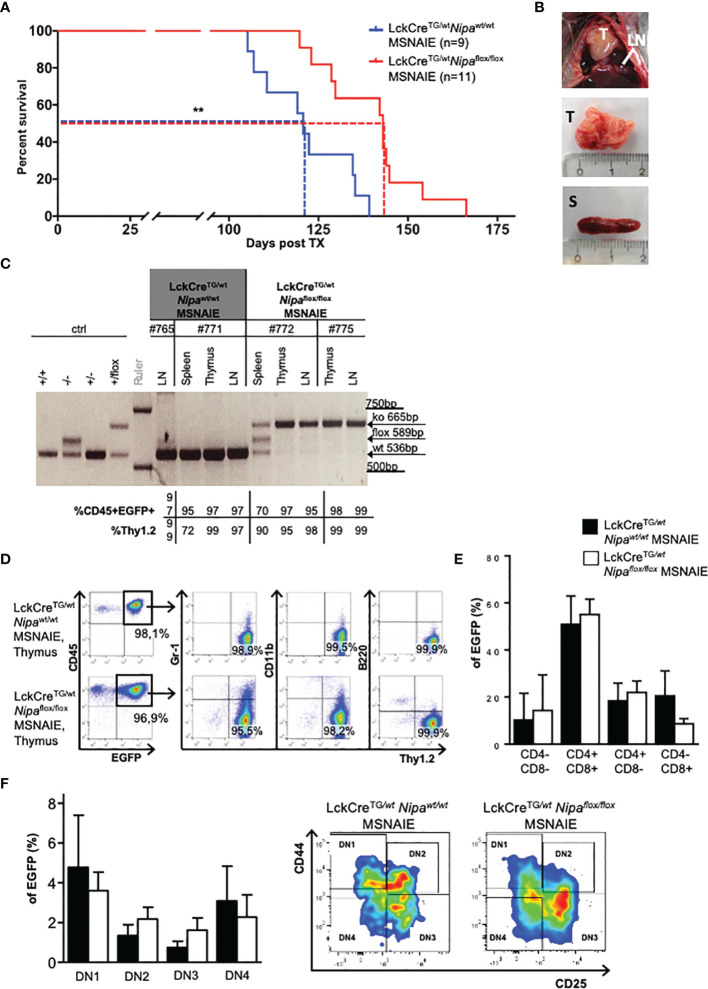
Deletion of *Nipa* delays lymphoma progression in an ALCL-like mouse model. **(A)** Kaplan–Meier survival curve of mice transplanted with *LckCre^TG/wt^Nipa^wt/wt^* MSNAIE and *LckCre^TG/wt^Nipa^flox/flox^* MSNAIE bone marrow. Median survival was 121 days (*Nipa^wt/wt^*, n = 9) versus 143 days (*Nipa^flox/flox^*, n = 11). Data from three independent transplantations was analyzed. **(B)** Representative images of infiltrated organs from mice transplanted with *LckCre^TG/wt^Nipa^flox/flox^* MSNAIE BMCs. LN, lymph node; T, thymus; S, spleen. **(C)** Gel electrophoresis showing lymphoma genotype of representative *LckCre^TG/wt^Nipa^wt/wt^* and *LckCre^TG/wt^Nipa^flox/flox^* MSNAIE transplanted mice in different lymphatic organs, correlated to EGFP-positivity and expression of T-cell markers. **(D)** Immunphenotyping of representative thymic lymphoma tissue determined by flow cytometry. **(E)** Mature T-cell distribution determined by flow cytometry for CD4 and CD8 in EGFP^+^ thymic cells of *LckCre^TG/wt^Nipa^wt/wt^* (n = 9) and *LckCre^TG/wt^Nipa^flox/flox^* MSNAIE (n = 16) transplanted mice. **(F)** DN T-cell subpopulations determined by flow cytometry due to CD44 and CD25 expression in EGFP^+^ thymic cells of *LckCre^TG/wt^Nipa^wt/wt^* (n = 8) and *LckCre^TG/wt^Nipa^flox/flox^* MSNAIE (n = 14) transplanted mice. Representative flow cytometry gating strategy on the right. *p < 0.05, **p < 0.01, ***p < 0.001. Data shown as mean +SD.

To establish a *Nipa*-deficient ALCL-like disease without “off-target” effects of *Nipa* deficiency, we used donor BMCs from *LckCre^TG/wt^Nipa^flox^* ^/flox^ mice for MSNAIE infection, thus restricting NPM-ALK expression and *Nipa* deletion to the identical T cells. Transplanted mice were monitored for clinical signs of disease, such as wasting, tachydyspnea, lymphadenopathy, and changes in the complete blood count. *LckCre^TG/wt^Nipa^flox/flox^* MSNAIE transplanted mice showed a later onset of disease with significantly prolonged survival. The median survival was significantly shorter (p = 0.002) with 121 days in *LckCre^TG/wt^Nipa^wt/wt^* MSNAIE transplanted mice compared to 143 days in *LckCre^TG/wt^Nipa^flox/flox^* (**Figure 3A**). At the final stage of the disease, mice were sacrificed and their organs harvested. Diseased mice presented with enlarged thymi of 470 mg on average, mediastinal and inguinal lymphadenopathy, and moderate splenomegaly independent of NIPA status (**Figure 3B** and **Supplementary Figure 2B**). Both bone marrow infiltration and leukocytosis were heterogeneous at comparable levels in both *Nipa* ^wt/wt^ and *Nipa* ^flox/flox^ transplanted mice (**Supplementary Figures 2C, D**). Correct *Nipa* deletion in lymphoma cells was validated by PCR analysis (**Figure 3C**) and lymphomas of *LckCre^TG/wt^Nipa^flox^* ^/flox^ MSNAIE transplanted recipients were further referred to as *Nipa* ^ko/ko^. As has previously been reported for wild-type lymphomas, immunophenotyping of *Nipa* ^ko/ko^ lymphoma cells showed a pure T-cell phenotype with negativity for myeloid and B-cell markers in the thymus, spleen, lymph nodes, peripheral blood, and bone marrow (**Figure 3D**) (Kreutmair et al., 2020a; Shoumariyeh et al., 2020). CD4/CD8 subpopulation analyses revealed a heterogeneous CD4/CD8 distribution in both *Nipa* ^ko/ko^ and *Nipa* ^wt/wt^ lymphomas (**Figure 3E**), which was, despite minor variations, similar in both groups with distinct CD4/CD8-double positive, CD4/CD8-double negative, and CD4- and CD8-single positive populations. Further analysis regarding the DN stages showed no significant difference between *Nipa* ^wt/wt^ and *Nipa* ^ko/ko^ lymphomas (**Figure 3F**). Our results therefore show that NIPA seems to play a significant role in NPM-ALK-induced lymphomagenesis but does not alter the disease immunophenotype of ALCL.

### NIPA is Associated With “Stem-Cell-Like” Features of T-Lymphocytes in ALCL-Like Lymphomas

**Figure 4:**
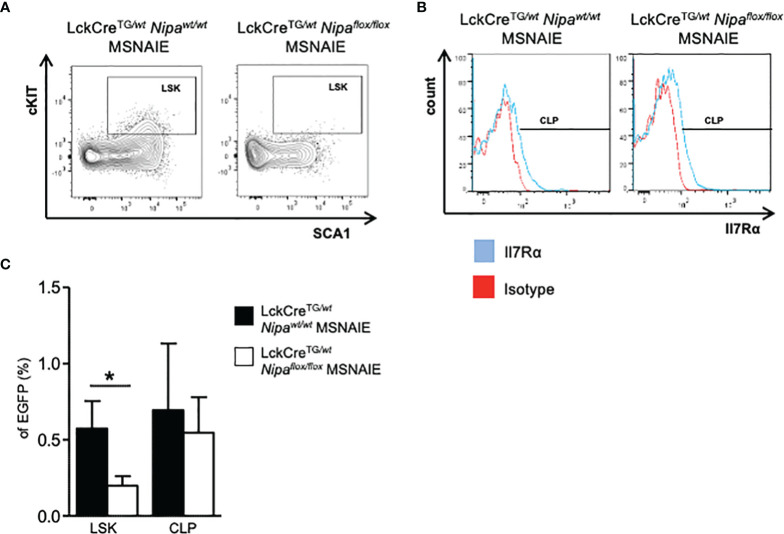
NIPA is associated with “stem-cell-like” features of T-lymphocytes in ALCL-like lymphomas. **(A)** Representative flow cytometry gating strategy for Lineage- (CD4^−^CD8^−^CD25^−^CD44^−^) SCA1+ cKIT+ subpopulation in EGFP^+^ thymic cells of *LckCre^TG/wt^Nipa^wt/wt^* and *LckCre^TG/wt^Nipa^flox/flox^* MSNAIE transplanted mice. EGFP^+^ Lineage-DN cells were stained for cKIT and SCA1. **(B)** Representative flow cytometry gating strategy for CLP subpopulation in EGFP^+^ thymic cells of *LckCre^TG/wt^Nipa^wt/wt^* and *LckCre^TG/wt^Nipa^flox/flox^* MSNAIE transplanted mice. EGFP^+^ Lin^−^ cKIT^low^ SCA1^low^ cells were stained for Il7Rα. **(C)** Proportion of subpopulations determined in **(A)** and **(B)** in EGFP^+^ thymic lymphoma cells of *LckCre^TG/wt^Nipa^wt/wt^* (n = 8) and *LckCre^TG/wt^Nipa^flox/flox^* (n = 12) MSNAIE transplanted mice. *p < 0.05, **p < 0.01, ***p < 0.001. Data shown as mean +SD.

Given the prolonged survival benefit in three different *Nipa*-deficient NPM-ALK-positive lymphoma mouse models, we investigated the regular activation of different oncogenic signaling pathways. Interestingly, in the presence of NPM-ALK, STAT3, AKT, and ERK1/2 demonstrated regular phosphorylation and therefore activation independent of NIPA (**Supplementary Figure 3A**). Thus, as it has been shown previously that *Nipa* deficiency significantly reduces hematopoietic stem cell frequency after replication stress due to impaired DNA damage repair followed by apoptosis (35), we analyzed thymic lymphomas of *Lck/Cre^TG/wt^Nipa^wt^* ^/wt^ and *Nipa* ^flox/flox^ MSNAIE transplanted mice for stem cell markers. We indeed found a small but distinct lymphoma subpopulation characterized by expression signatures Lineage-negative (CD4^−^CD8^−^CD25^−^CD44^−^), cKIT, and SCA1-positive, which was significantly lower in *Nipa^ko^* ^/ko^ lymphoma cells, being only one-third of *Nipa* ^wt/wt^ cells (0.19% vs. 0.57%, p = 0.04, **Figures 4A, C** and **Supplementary Figure 3B**). Common lymphoid progenitors, measured by positivity for Il7Rα, demonstrated no significant difference in the dependency of NIPA (**Figures 4B, C**).

Taken together, the *Nipa*-deficient ALCL-like mouse model demonstrates a crucial role of NIPA in primary ALCL-like lymphomas. The *Nipa-*deleted lymphomas showed a reduced frequency of lymphoma cells expressing stemness markers, which may thereby contribute to the decelerated course of disease.

## Discussion

NIPA has recently been described as a crucial regulator of mitotic entry and bone marrow failure (35, 42), but has not yet been analyzed in NPM-ALK-induced lymphomas, where it was initially found as an interaction partner of NPM-ALK. Ouyang et al. have shown that NIPA interacts with NPM-ALK in a kinase-dependent manner and can protect Ba/F3 cells from apoptosis (31). The results of this study identify NIPA as a relevant player in efficient NPM-ALK mediated lymphomagenesis. In our study, *in vitro* assays with different cell lines showed that proliferation and transformation of NPM-ALK-positive MEFs, Ba/F3, and Karpas299 cells were significantly impaired upon NIPA deficiency or downregulation, which was not due to the already described cell cycle-dependent function of NIPA. *In vivo* experiments in different murine NPM-ALK-driven tumor models extended these results and demonstrated that transplantation of NPM-ALK-positive *Nipa^ko/ko^* BMCs and BM T-lineage restriction of *Nipa* deletion and NPM-ALK expression led to significantly prolonged survival compared to *Nipa^wt/wt^* transplanted animals. The absence of immunophenotype changes may reflect the known functions of the protein as a regulator of the cell cycle and DNA damage repair rather than cell differentiation.

Based on the prolonged survival of recipient mice transplanted with *Nipa-*depleted cells in three different BM transplantation models and regular oncogenic signaling pathways independent of NIPA, we hypothesized that NIPA is relevant for ALCL lymphoma initiation. Recent studies of *Nipa^ko/ko^* mice demonstrated reduced numbers and function of hematopoietic stem cells (HSCs). *Nipa*-deficient HSCs showed cell-intrinsic defects leading to reduced proliferation capacity, accumulation of DNA damage, and cell death due to impaired DNA damage/FA/BRCA pathway. Furthermore, aged or replication-stressed *Nipa-*deficient animals developed bone marrow aplasia (35). We found a lymphoma subpopulation characterized by the stem cell markers Lineage-cKIT^+^ SCA1^+^ being significantly reduced in *Nipa^ko/ko^* lymphomas. This may influence the observed prolonged disease latency by regulating the lymphoma stem cell reserve. In the past years, the so-called “cancer stem cell theory” has been described for various hematological and solid malignancies. According to this theory, only a small percentage of cells in an overall heterogeneous malignancy show tumor promoting characteristics, such as specific surface markers, gene expression profiles, or the ability to generate identical xenografts (14, 43, 44). Various studies have explored the originating cells of NPM-ALK-positive ALCL, and there is accumulating evidence that lymphoma initiation starts in a primitive cell population at an undifferentiated T cell or even HSC-like level—genetically reprogrammed and independent of their phenotype (14). Moti et al. have identified a side population in ALCL that proliferated more than the main population and could therefore give rise to xenografts (45). This side population expressed a gene profile similar to that of early thymic progenitor cells, supporting the hypothesis of a stem cell origin. Yet regarding the immunophenotype, the ability to form xenografts was independent from the presence of hematopoietic stem cell markers (45). A different study showed the importance of the embryonic stem cell factor Sox2 for ALCL xenograft growth, a hint towards the importance of progenitor cells for tumor propagation (46).

In the case of *Nipa* deficiency, the number of both healthy hematopoietic stem cells in aged mice and stemness marker-expressing lymphoma cells in the NPM-ALK-positive disease is reduced. In healthy HSCs, NIPA plays a crucial role in DNA damage repair as a regulator of FANCD2, the key player in the Fanconi anemia pathway, rather than regulating the cell cycle itself. Thus, replication stress is a major risk factor for the *Nipa-*deficient stem cell pool. Taking into consideration that cancer stem cells necessarily undergo substantial replication stress at the time of lymphoma initiation and development, one may hypothesize that NIPA regulates this phase of lymphatic disease. Thus, NIPA may act at the level of the ALCL “stemness” cell population, but it might as well be that it positively influences the malignant transformation in general by protecting cells from apoptosis. However, *in vitro* analysis of Mig^NPM-ALK^ transduced Ba/F3 and Karpas299 cells transfected with either pLMP^miRmNIPA^ or pLMP^miRctrl^ did not demonstrate major differences in apoptosis (data not shown).

It would be interesting to see if a phosphorylation-deficient mutant of *Nipa* at the recently identified serin/threonin sites relevant for NIPA-NPM-ALK binding changed the course of the ALCL-like disease. Taking into account that a phosphorylation-deficient *Nipa* has negatively influenced proliferation upon NPM-ALK expression *in vitro* (36), it is possible that those sites also play a crucial role *in vivo*. One could furthermore hypothesize that this phosphorylation-deficient *Nipa* mutant also led to impaired DNA damage/FA/BRCA pathway or deregulation of mitotic entry in NPM-ALK-driven lymphomagenesis. Further characterization of the NIPA/NPM-ALK interaction in *in vivo* mouse models and human ALCL might be necessary to elucidate the exact underlying mechanistic pathways.

In summary, we could show that NIPA is essential for effective initiation of NPM-ALK-driven ALCL-like disease in a clinically relevant mouse model, while it seems dispensable for the lymphoma immunophenotype. Highlighting the importance of the NIPA/NPM-ALK axis in lymphoma development, clinical assessment of NIPA may provide a basis for future therapeutic approaches.

## Data Availability Statement

The raw data supporting the conclusions of this article will be made available by the authors, without undue reservation.

## Ethics Statement

The animal study was reviewed and approved by the Regierungspräsidium Freiburg, 79095 Freiburg im Breisgau.

## Author Contributions

Conceptualization, JD and ALI. Methodology, SK, CK, CA-L, CM, and ALI. Investigation, SK (**Figures 3**, **4**), LJL (**Figures 1**, **3**), CK (**Figure 3**), CA-L (**Figure 2**), VS (**Figure 3**), and TM (**Figure 3**). Data curation, SK (**Figures 3** and **4**), LJL (**Figures 1** and **3**), CK (**Figure 3**), CA-L (**Figure 2**), and VS (**Figure 3**). Formal analysis, SK (**Figures 3**, **4**), LJL (**Figures 1**, **3**), CK (**Figure 3**), and CA-L (**Figure 2**). Resources, ALI. Writing—original draft preparation, SK and LJL. Writing—review and editing, SK, LJL, CK, CA-L, SY, VS, TM, AM-R, CY, SPG, CM, JD, and ALI. Visualization, SK and LJL. Supervision, SK, CK, and ALI. Project administration, SK, CK, and ALI. Funding acquisition, ALI and JD. All authors listed have made a substantial, direct, and intellectual contribution to the work and approved it for publication.

## Funding

SK was supported by the SUCCESS-Program of the University Hospital Freiburg and the Berta-Ottenstein-Program of the University of Freiburg. LL was supported by a scholarship from DGHO/José Carreras Foundation. SY was supported by a scholarship from DGHO/ Sieglinde Welker Foundation. This work was supported by a grant from MSCA-ITN-2015-ETN Alkatras to ALI and JD and MSCA-ITN-2022-ETN FANTOM to ALI and grants from the DFG to ALI (SFB 1479) and JD (FOR 2033 B1). ALI was supported by an ACSS from the DGIM and the Mildred-Scheel-Professorship Program by the German Cancer Aid (70114112).

## Conflict of Interest

The authors declare that the research was conducted in the absence of any commercial or financial relationships that could be construed as a potential conflict of interest.

## Publisher’s Note

All claims expressed in this article are solely those of the authors and do not necessarily represent those of their affiliated organizations, or those of the publisher, the editors and the reviewers. Any product that may be evaluated in this article, or claim that may be made by its manufacturer, is not guaranteed or endorsed by the publisher.
