# Supplementary material for: NIPA (Nuclear Interaction Partner of ALK) Is Crucial for Effective NPM-ALK Mediated Lymphomagenesis
Source: Front Oncol. 2022 May 13;12:875117. doi: 10.3389/fonc.2022.875117 (PMC9137267; doi:10.3389/fonc.2022.875117)
Supplement: Supplementary file 1 [file DataSheet_1.pdf]

frontiers

Supplementary Material

1.1 Supplementary Figure 1

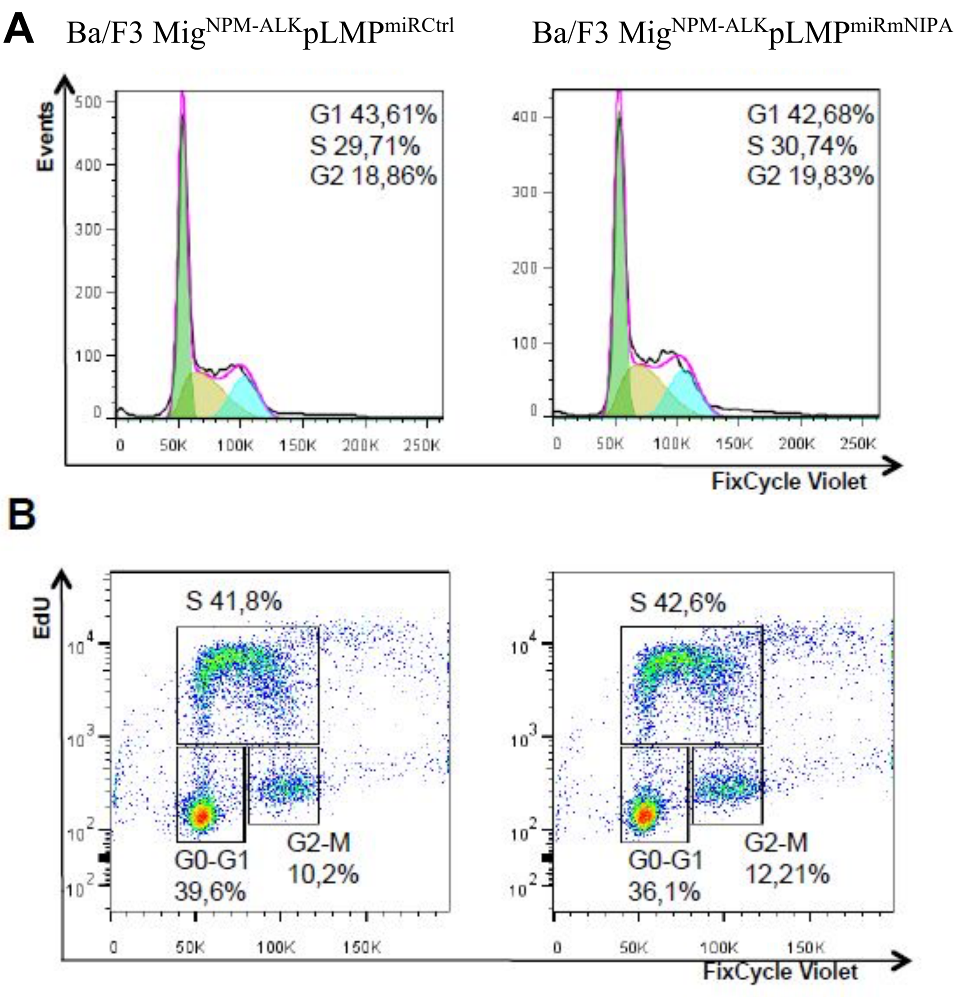

Supplementary Figure 1.

A) Static cell cycle analysis (Dean-Jett-Fox calculation) and

B) Dynamic cell cycle analysis of Ba/F3-cells retrovirally infected with Mig$^{NPM\text{-}ALK}$ and pLMP^{miRmNIPA} or pLMP^{miRctrl}, after 1 h incubation with EdU and staining with FxCycle Violet.

1.2 Supplementary Figure 2

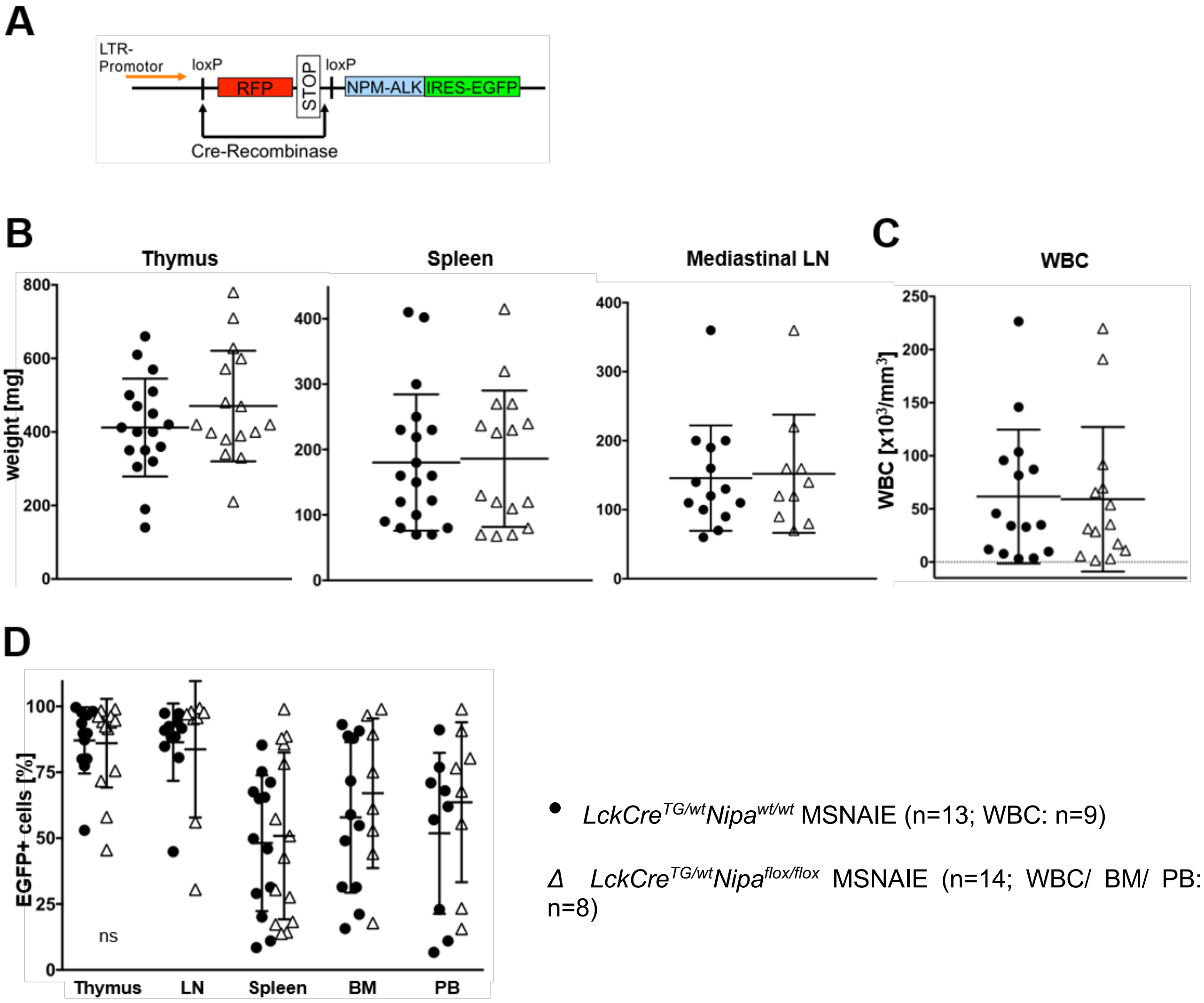

Supplementary Figure 2.

A) Retroviral construct MSNAIE (MSCV-STOP-NPM-ALK-IRES-EGFP).

B) Organ weight of spleen, thymus and mediastinal lymph nodes and white blood count of mice transplanted with *LckCre$^{TG/wt}$Nipa$^{wt/wt}$* MSNAIE and *LckCre$^{TG/wt}$Nipa$^{flox/flox}$* MSNAIE bone marrow at death. Mean weight shown of *LckCre$^{TG/wt}$Nipa$^{wt/wt}$* MSNAIE thymus (n=18), spleen (n=19), mediastinal LNs (n=14), WBC (n=15) and *LckCre$^{TG/wt}$Nipa$^{flox/flox}$* MSNAIE thymus (n=16), spleen (n=16), mediastinal LNs (n=10), WBC (n-=4). p>0.05.

C) Infiltration of lymphatic organs, peripheral blood and bone marrow measured by EGFP-positivity in mice transplanted with *LckCre$^{TG/wt}$Nipa$^{wt/wt}$* MSNAIE and *LckCre$^{TG/wt}$Nipa$^{flox/flox}$* MSNAIE bone marrow at death. Frequency shown of *LckCre$^{TG/wt}$Nipa$^{wt/wt}$* MSNAIE thymus (n=13), mediastinal LNs (n=11), spleen (n=13) and *LckCre$^{TG/wt}$Nipa$^{flox/flox}$* MSNAIE thymus (n=14), mediastinal LNs (n=8), spleen (n=14), bone marrow (n=8), peripheral blood (n=8), bone marrow (n=12), peripheral blood (n=9). p>0.05.

1.3 Supplementary Figure 3

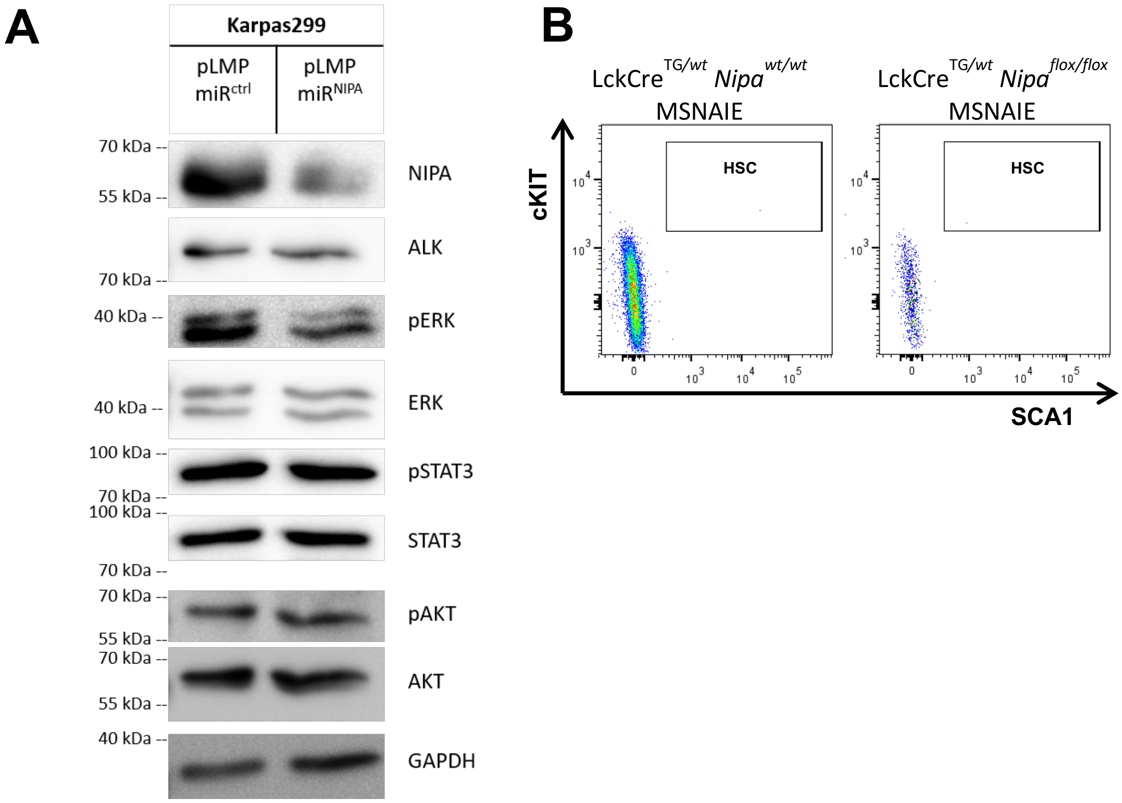

Supplementary Figure 3.

A) Western blot analysis for STAT3, AKT, ERK1/2 and their phosphorylation status. Primary Mefs (*Nipa*$^{ko/ko}$ MEFs retrovirally infected with pBABE-puroR^{Nipa} or pBABE-puroR^{empty} vector and Mig$^{NPM\text{-}ALK}$ or Mig^{empty} vector), Karpas299 (retrovirally infected with pLMP^{miRhNIPA} or pLMP^{miRctrl}) and lymphoma mouse tissue (Thymus of *LckCre*$^{TG/wt}$*Nipa*$^{wt/wt}$ MSNAIE and *LckCre*$^{TG/wt}$*Nipa*$^{flox/flox}$ MSNAIE transplanted mice) was used for analysis.

B) Representative flow cytometry isotype controls for "LSK" subpopulation in EGFP$^+$ thymic cells of *LckCre*$^{TG/wt}$*Nipa*$^{wt/wt}$ and *LckCre*$^{TG/wt}$*Nipa*$^{foxl/flox}$ MSNAIE transplanted mice. EGFP$^+$ Lineage$^-$ DN cells are stained for cKIT and SCA1. *p<0.05; **p<0.01; ***p<0.001. Data are represented as mean ± SD.
